# Cu-bearing stainless steel reduces cytotoxicity and crystals adhesion after ureteral epithelial cells exposing to calcium oxalate monohydrate

**DOI:** 10.1038/s41598-018-32388-0

**Published:** 2018-09-20

**Authors:** Zhiqiang Cao, Jing Zhao, Ke Yang

**Affiliations:** 1 General Hospital of Shenyang Military Region, Shenyang, 110840 China; 2 Institute of Metal Research, Chinese Academy of Sciences, Shenyang, 110016 China

## Abstract

Calcium oxalate monohydrate (COM), which is the main component of encrustation, may result in cell membrane injury. In addition, cellular damage is suggested to be the primary event attributing to COM crystal binding. To study the interaction between cells and crystals after incubating with a Cu-bearing stainless steel (316L-Cu SS), MTS and flow cytometric analyses were used to assess the cellular responses. The results confirmed that 316L-Cu SS could inhibit cytotoxicity and cellular apoptosis of ureteral epithelial cells (UECs) after COM treatment. Furthermore, molecular expressions of Cu/Zn superoxide dismutase (CuZnSOD), which were evaluated by western blot analysis and real-time quantitative PCR (qPCR), indicated that 316L-Cu SS could inhibit the oxidative stress attributing to up-regulating of CuZnSOD. Moreover, the crystal adhesion cytokine CD44 was examined with western blot and qPCR, and the corresponding hyaluronic (HA) secreted into the medium was measured by enzyme-linked immunosorbent assay (ELISA). All results were confirmed that the expressions of cells cultured with 316L-Cu SS were down-regulated, demonstrating the inhibitory performance of 316L-Cu SS against crystal adhesion.

## Introduction

Ureteral stents, catheters, and other urological implants are easily affected by encrustation, which is predominantly composed of precipitated calcium salts^1,2^. Calcium oxalate monohydrate (COM) is the principal crystalline compound found in the stone mass, with a frequency up to 77.5%^3,4^. Generally, human urine promotes the nucleation of COM crystals under suitable conditions^5^. In addition to crystal growth and aggregation, the adhesion of COM crystals to epithelial cells is another key factor influencing concretion^6,7^. The COM has strong ability to bind at cell surfaces and thus becomes an aggregation center for stone formation^8^.

A high concentration of oxalate contributes to COM precipitation. Oxalate and/or COM induce cellular oxidative stress in the kidneys with increasing free radical production, which creates a harmful environment for macromolecules and results in cell damage and even apoptosis or cell death^9–12^. Moreover, different types crystals generate different amounts of ROS, but to be sure, they all show toxicity to cells^13^. Copper and zinc superoxide dismutase (CuZnSOD), which is one member of a family of antioxidant enzymes, is a copper- and zinc-containing homodimer that catalyses the disproportion of superoxide to hydrogen peroxide and dioxygen and scavenges superoxide, thus providing the cell with a major defence barrier against oxygen toxicity^14,15^. According to previous reports, continual supply of dietary copper is needed to sustain a steady-state level of functional CuZnSOD in many tissues^16^. Accordingly, appropriately adding copper to cells may protect them from injury.

In general, the cell injury caused by COM alters gene expression and protein production of various chemoattractants and urinary macromolecules^17^, which not only play vital roles in the inflammatory reaction and cellular signalling pathways contributing to stone formation, but also are involved in regulating crystal nucleation, growth and deposition^18^. Hyaluronan (HA) and its cell surface receptor, CD44, were found to play an important role in COM crystal binding during wound healing caused by stent implantation^19^. HA is a high-molecular-mass polysaccharide in many tissues and performs a wide range of biological functions *in vivo*^20^. It has been identified as a major crystal-binding molecule on the surface of human renal tubular cells and is abundantly present as the main component of the renal inner medullary interstitium. Various inflammatory disease states may up-regulate HA^21,22^. Similarly, the transmembrane protein CD44, which is a cell surface receptor for HA, is also up-regulated during inflammation^23,24^. Its expression is accompanied by an increased expression of its ligand HA, both of which induce crystal binding.

In the previous study, a Cu-bearing stainless steel (316L-Cu SS) has been proved to satisfy the basic requirements for ureteral stent material, demonstrating special anti-infection properties^25^. In addition, we also found another positive effect, namely, *in vitro* reduction of encrustation free of cells^26^. Therefore, the aim of this study was to further investigate the cellular response to COM crystal-induced toxicity in ureteral epithelial cells (UECs), with the expectation of providing more evidence to support the application of 316L-Cu SS as a novel ureteral stent material in clinic.

## Results

### 316L-Cu SS promoting UECs viability

**Figure 1:**
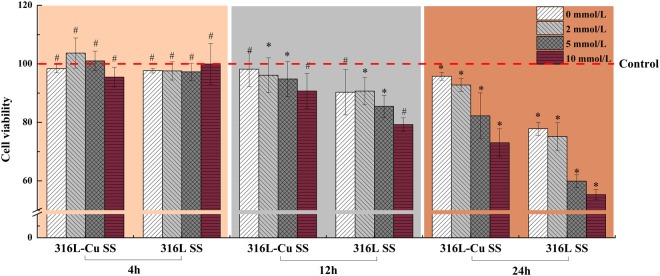
Viability of UECs incubated with different extracts plus various concentrations of oxalate (0, 2, 5 or 10 mM) and COM (200 μg/ml) for 4 h, 12 h and 24 h, respectively. The cells viability was determined using a MTS kit according to the product protocol. The data shown are the mean and standard deviation (SD) (N = 5). *Indicates a significant difference at *p* < 0.05 (*p < 0.05), and ^#^indicates no significant difference at *p* > 0.05 (^*#*^*p* > 0.05) compared to 316L SS incubated for the same time.

Cells exposed to oxalate (0, 2, 5 or 10 mM) plus COM crystals (200 μg/ml) for different time intervals (4, 12 and 24 h) were determined. As shown in Fig. 1, the relative growth rate of UECs incubated with 316L-Cu SS or 316L SS for 4 h was similar to that of the control, which demonstrated that oxalate and COM did not significantly influence cells at the beginning of incubation. When the incubation time was increased to 12 h, the oxalate and COM crystals showed concentration-dependent toxicity on UECs. Furthermore, the toxicity to cells, whether they were incubated with 316L-Cu SS or 316L SS for 24 h, increased dramatically when the concentration of oxalate was over 5 mM. However, the viability of the cells incubated with 316L-Cu SS was more activated than that of 316L SS, implying that Cu^2+^ ions play a vital role in promoting cell viability.

### 316L-Cu SS inhibiting UECs apoptosis

**Figure 2:**
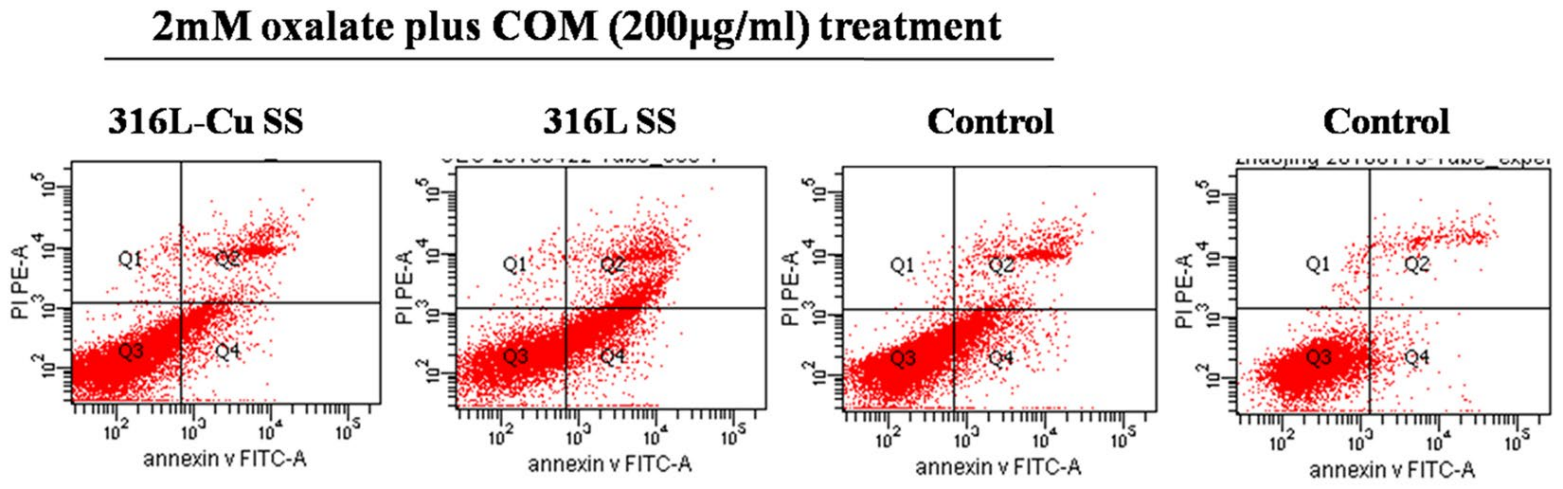
Flow cytometric analyses of the apoptosis of UECs. Cells were seeded on the samples and exposed to oxalate (2 mM) plus COM (200 μg/ml).

To more clearly study the effect of 316L-Cu SS on UECs, a treatment of 2 mM oxalate plus COM (200 μg/ml) was selected because the relative growth rates of cells incubated with 316L-Cu SS were all above 85%, which is considered non-toxic to cells according to ISO 10993–5:2009 guidelines. Figure 2 demonstrates the scatter plots of UECs detected by flow cytometry. The second and forth quadrant represent the area of cells apoptosis at the late stage and early stage of incubating, respectively. Meanwhile, the density of the dots in the second and forth quadrant correspond to the amount of the cells apoptosis. It can be seen that the amount of apoptotic UECs cultured on the surface of 316L-Cu SS was nearly the same as control after exposing to 2 mM oxalate plus COM (200 μg/ml). However, it was significantly lower than those on the 316L SS. The apoptotic rate measured from the dots density is listed in Table 1. It was indicating that metal ions released from 316L SS would effect on the viability of UECs, whereas Cu^2+^ ions from 316L-Cu SS could maintain the viability of UECs.

**Table 1:** Apoptosis rates of UECs incubated on samples and plates after different treatments.

	2 mM oxalate plus COM treatment			Control
	316L-Cu SS	316L SS	Control	
Early apoptosis rates	17.47 ± 1.42^b^	28.67 ± 1.07^a^	19.00 ± 2.62^b^	3.10 ± 0.21^c^
Late apoptosis rates	9.03 ± 0.61^b^	14.33 ± 1.18^a^	9.43 ± 1.11^b^	3.33 ± 0.71^c^

Data are presented as the mean ± SD. A significant difference at *p* < 0.05 is shown by different letters (a, b and c). Early apoptosis rates and late apoptosis rates were compared.

### 316L-Cu SS activating CuZnSOD vitality and inhibiting ROS release

**Figure 3:**
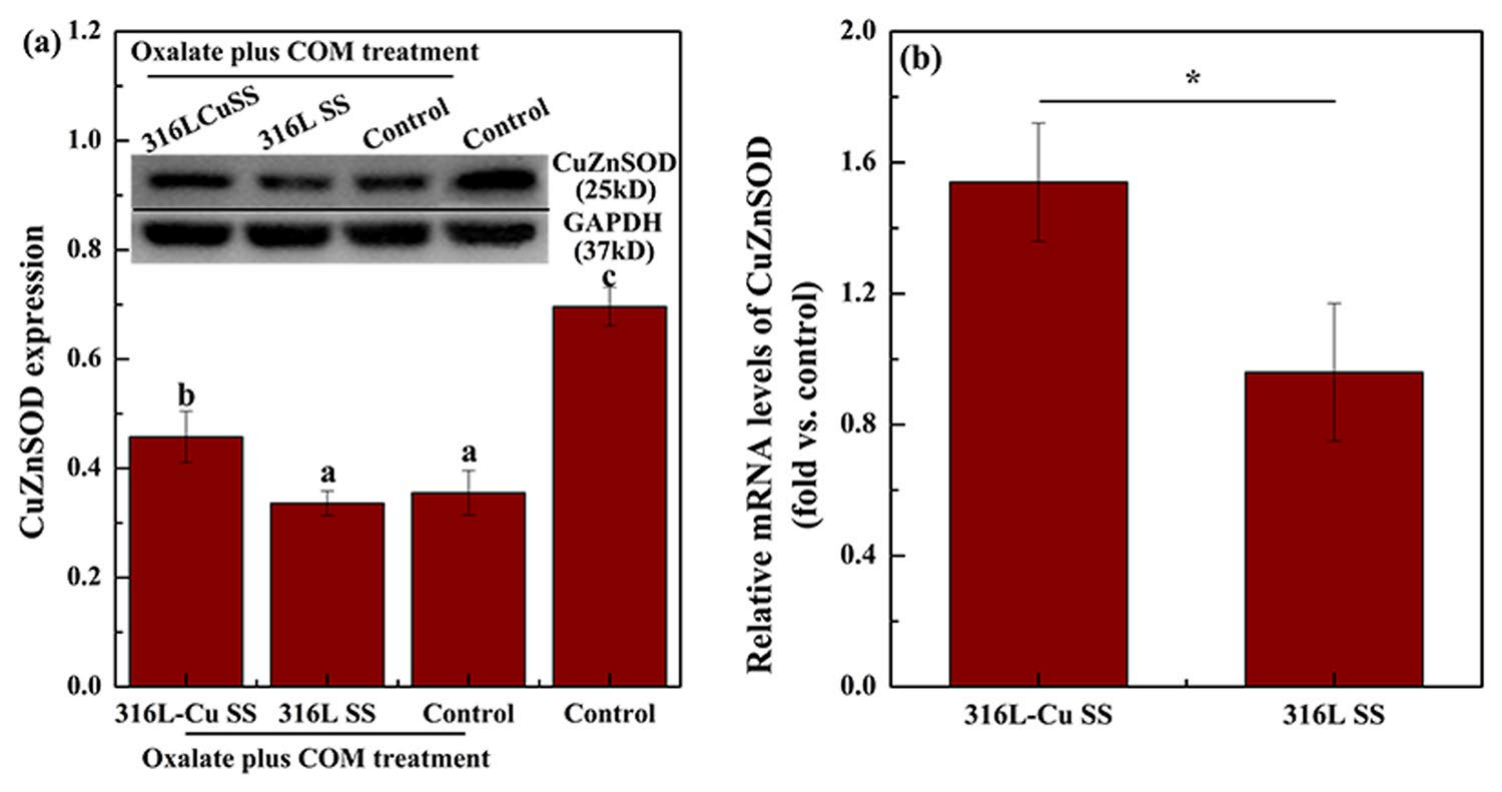
316L-Cu SS activated the expression of CuZnSOD of UECs. (**a**) Expression of CuZnSOD and (**b**) qPCR analysis of the CuZnSOD-related gene expression for UECs. Cells were seeded on the surfaces of 316L-Cu SS and 316L SS. DMEM containing oxalate (2 mM) and COM (200 μg/ml) was used to induce cell injury. After incubating for 24 h, the cells were harvested and used for western blot analysis. Anti-GAPDH antibody was used to evaluate equal protein loading on the blot. The density of the bands and relative mRNA levels were expressed as the fold difference from the housekeeping protein and gene, respectively. Then, the levels were calculated relative to the control, which was given the same treatment. Values are presented as the mean ± SD (N = 3). A significant difference at *p* < 0.05 is shown with different letters *p < 0.05. Intact western blot results can be seen in Supplementary information.

**Figure 4:**
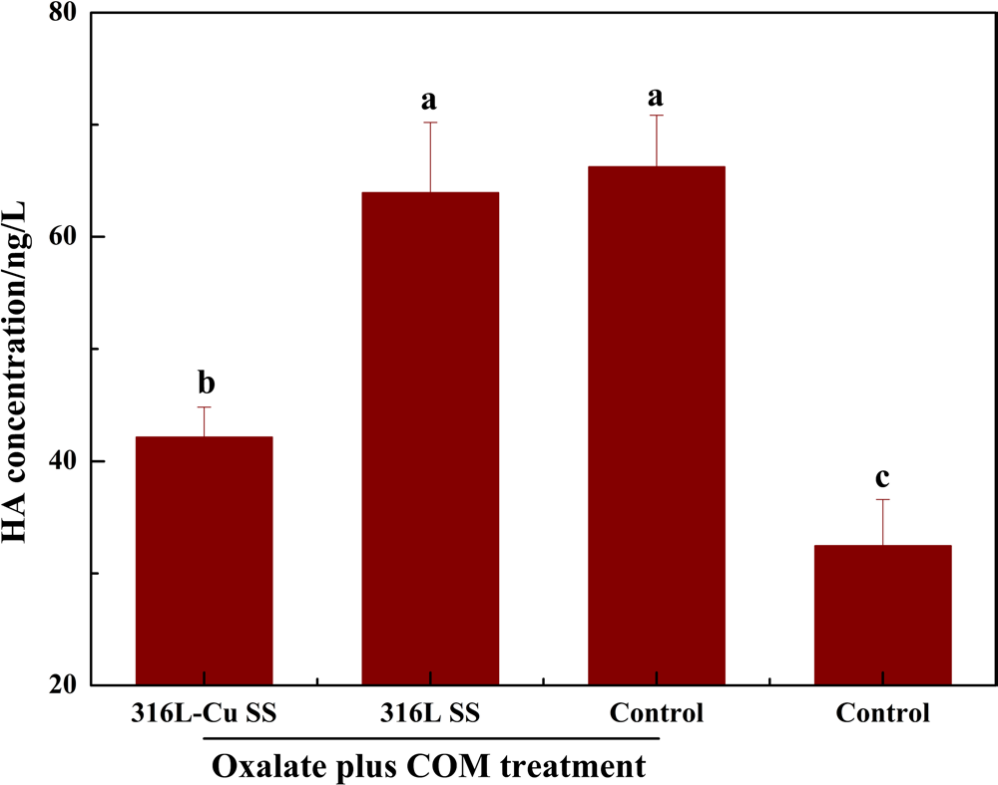
Concentrations of ROS after different treatments of UECs for 24 h. Data are presented the as mean ± SD. A significant difference at *p* < 0.05 is shown with different letters.

CuZnSOD expression, which was used to evaluate cell viability, was examined by western blot analysis and qPCR. Figure 3(a) shows that after COM treatment, the CuZnSOD expression of the control was dramatically lower than that of the control without COM treatment. The CuZnSOD level of 316L-Cu SS-administered UECs was significantly higher than that of 316L SS-administered UECs. Furthermore, qPCR was used to study the CuZnSOD-related gene expression as plotted in Fig. 3(b). In the cytosol fraction of UECs, Student’s t-test showed that 316L-Cu SS positively affected CuZnSOD enzymatic activity, whereas 316L SS maintained nearly the same value as the control, suggesting that 316L-Cu SS had an activating effect on CuZnSOD vitality. In addition, the ELISA results of reactive oxygen species (ROS) give a supplement of CuZnSOD as depicted in Fig. 4. Specifically, UECs cultured with 316L-Cu SS released a lower level of ROS, illustrating that Cu^2+^ was a key element in blunting oxidative stress.

### 316L-Cu SS attenuating CD44 and HA expression

**Figure 5:**
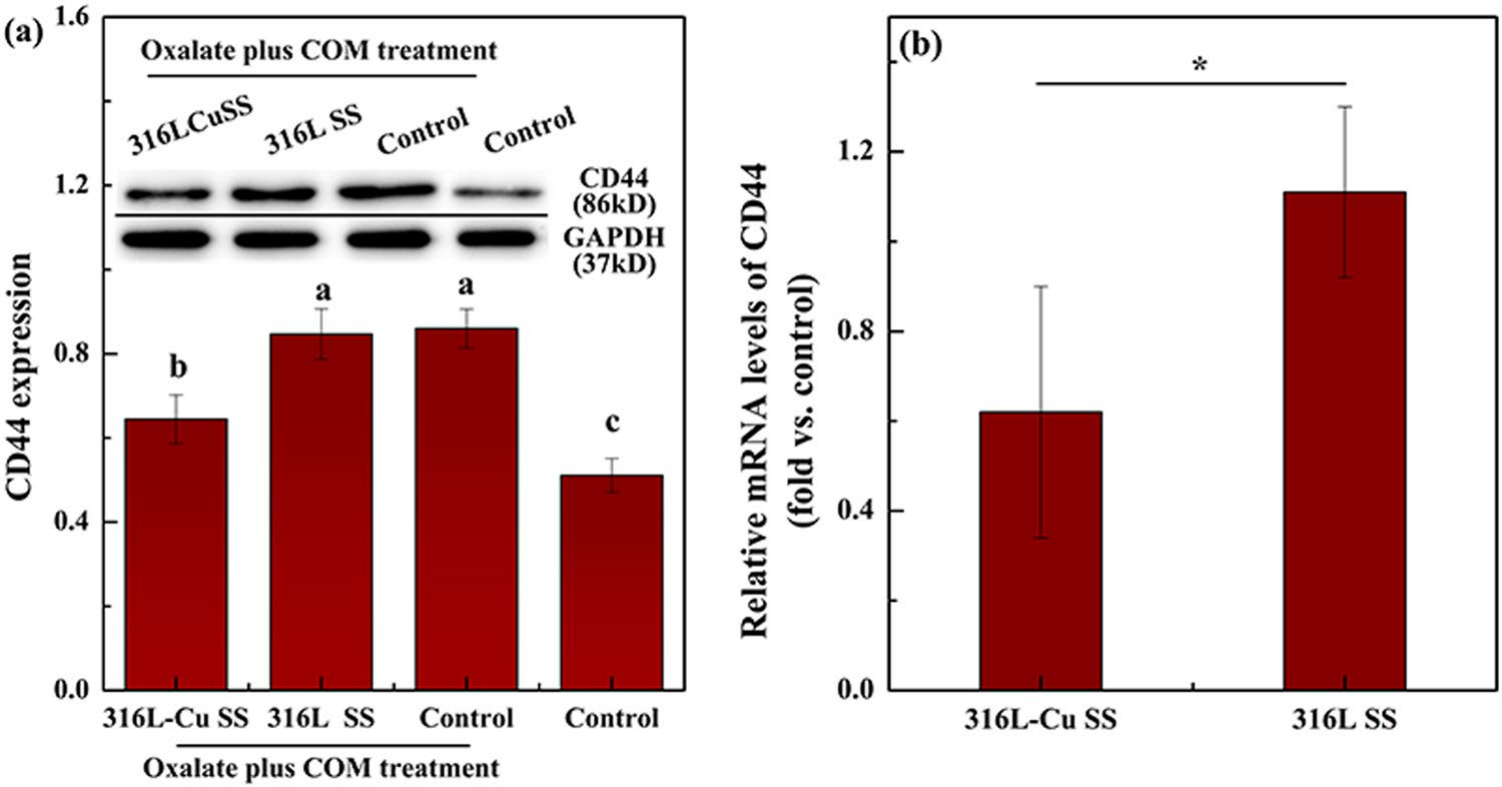
316L-Cu SS attenuates the expression of CD44. (**a**) Western blot analysis and (**b**) relative mRNA expression gene transcription of CD44. A significant difference at *p* < 0.05 is shown with different letters *p < 0.05. Intact western blot results can be seen in Supplementary information.

**Figure 6:**
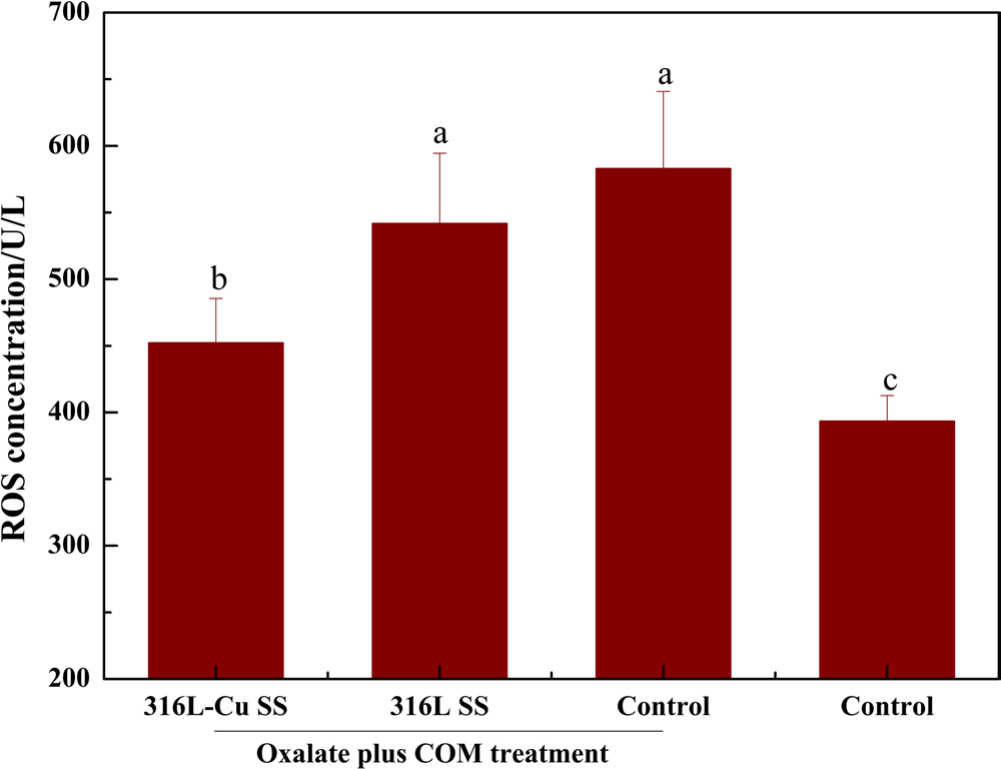
HA secretion into the culture medium after incubating for 24 h. A significant difference at *p* < 0.05 is shown with different letters.

CD44 expression of UECs cultured with 316L-Cu SS for 24 h after oxalate plus COM treatment was significantly reduced, whereas with 316L SS, it was close to the control as presented in Fig. 5(a). Moreover, Fig. 5(b) depicts that the relative mRNA expression level of CD44 obviously decreased after exposure to 316L-Cu SS. In addition, the level of HA secretion into the medium was measured by ELISA. The supernatant incubated with 316L-Cu SS was found to secrete less HA than that with 316L SS, as plotted in Fig. 6, which revealed a uniform trend towards CD44. It was confirmed that 316L-Cu SS down-regulated both CD44 and HA, which might further inhibit crystal deposition and/or formation.

## Discussions

Cellular injury is one of the most important factors promoting encrustation. Currently, the prevalently used polymeric double J ureteral stent breaks down the ureter through constant friction in the clinic. In addition, it tends to adhere to crystals at a position of cellular injury and acts as nuclei for gradual encrustation^27^. Although many investigators recognize that oxalate precipitates as COM in the primary urine, which is attributed to its poor solubility, Khan^28^ believed that crystals binding to cells may also contribute to encrustation. It can be supposed that a prospective ureteral stent material, which reduces the injury caused by crystals after implantation, may solve this problem. 316L-Cu SS was shown to be a novel material to potentially relieve complications, including infection, fibrosis and encrustation, due to its continuous release of a trace amount of Cu^2+^ ions, which affect cell function during the period of service^26^. In addition, the current results imply that 316L-Cu SS reduces the cytotoxicity induced by oxalate and COM, while inhibiting crystals from binding to cells.

A special cycle accelerating stone formation in a urinary system has been confirmed^29^. Adhesion of COM crystals will induce the injury and apoptosis of renal tubular epithelial cells, and vice versa, COM-induced cellular injury can facilitate COM crystal adhesion^30^. Thus, UECs are more easily injured by crystal-cell interactions. As a result of pre-existing cell injury, changes in the cellular surface properties accompany crystal adhesion, whereas normal cells exhibit almost no concretion^31^. Besides, oxalate at a high concentration is also harmful to cells due to its direct interaction with cells to produce COM crystals. Oxalate and COM together are even more harmful due to their synergistic action^32^. Both animal models of *in vivo* hyperoxaluria and *in vitro* cell culture studies demonstrated that exposure to high levels of oxalate and COM might injure epithelial cells and trigger serial responses related to stone formation^33^. 316L-Cu SS promoted the viability of cells exposed to oxalate plus COM by continuously releasing of Cu^2+^ ions, which finally contributed to inhibiting the encrustation formation. It turned out that the toxicity of cells was mainly influenced by oxidative stress induced by oxalate and COM. After adhesion of COM, the integrity and the polarity of cytomembrane were changed, resulting in the release of ROS, such as superoxide and H_2_O_2_ and then inducing apoptosis^34^. If additional ROS production could not be dealt with or endogenous antioxidant defenses were depleted after exposure to COM, a reduction in the activity of antioxidant enzymes and even cell death would occur^28,35,36^. CuZnSOD is universally thought to resist ROS due to its anti-oxidative property. Over-production of CuZnSOD protects cells from injury by catalysing disproportion of ROS and eliminating ROS. Cu, which acts as a cofactor in the synthesis of CuZnSOD, promotes the viability of cells^16^. The steady release of trace amounts of Cu^2+^ ions from 316L-Cu SS up-regulated CuZnSOD of UECs results in hindering ROS production. Furthermore, the bio-function of decreasing injury caused by crystals will finally inhibit the cell toxicity and apoptosis.

HA and its mutual cell surface receptor CD44 play a vital role in the binding of COM crystals after wound healing, i.e., cell injury. HA accumulates in wounded tissue shortly after injury and forms loose hydrated matrices that allow cell division and migration. Several results have identified HA as a major crystal-binding factor based on the following: (1) crystals adhere to HA-expressing cells rather than those no longer expressing HA and (2) crystal binding could be decreased by an enzyme with the special ability of digesting HA^37^. Therefore, cell repair or regeneration resulting in decreasing HA secretion after injury is an effective method of inhibiting crystal binding. In fact, the biological activity of HA predominantly depends on its interaction with CD44^38^, which primarily function in transporting messages between cells. Generally, CD44 is minimally expressed in the kidney, ureter and urethra. Nevertheless, once these organs are injured to different degrees, expression of CD44 dramatically up-regulates^21^. Verkoelen^39^ confirmed that expressions of HA and CD44 increased significantly after the cells were stimulated by COM. Phosphatidylserine (PS), which supports COM crystal attachment to the renal epithelial cells, promoted crystal adherence when its content was increased or redistributed it from the inner to the outer leaflet of the membrane^40^. The change in the membrane structure provides an effective locus for crystal attachment. More remarkably, the modification of cell membrane phospholipids is activated by ROS, which results in crystal binding. Thus, HA and CD44 up-regulation can also be attributed to the release of ROS, which further suggests the interaction between crystal binding cytokines and cell injury. 316L-Cu SS was proven to activate CuZnSOD, accompanied by decreasing ROS. Accordingly, Cu^2+^ ions released from the material can be concluded to significantly inhibit crystal attachment.

In summary, the data from the present study demonstrated that 316L-Cu SS, which is a novel ureteral stent material, can potentially inhibit the cytotoxicity produced by crystal injury. The crystal adherent binding player cytokines CD44 and their ligand HA are negatively regulated with a decreasing level of ROS, which hinder the pathogenesis of injury disorders. Cu^2+^ ions contribute to the anti-oxidative property by activating CuZnSOD viability, which finally mitigates crystal adherence. In addition, the continuous release of Cu^2+^ ions from 316L-Cu SS will contribute to the CuZnSOD viability, which steadily reduces toxicity and encrustation.

## Conclusion

This study showed positive effects of Cu-bearing stainless steel, 316L-Cu SS, on inhibiting encrustation formation. Mainly because of decreased the cytotoxicity of UECs, inhibited crystal adhesion and it has potential to be used as a novel ureteral stent material.

## Methods

### Materials preparation

The experimental material used in this study was an antimicrobial 316L type Cu-bearing stainless steel (316L-Cu SS) fabricated by adding 4.5%Cu into 316L stainless steel, with normal chemical composition of Fe-19Cr-14Ni-2Mo-4.5Cu, wt.%. Medical grade 316L stainless steel (316L SS) was served as the control. All the forged experimental steels were solution treated at 1050 °C for 0.5 h followed by a water quench, and then were aged at 700 °C for 6 h. All the samples were cut into sizes of φ10 mm × 1 mm and mechanically polished sequentially with SiC papers from 180 to 2000 grit. The samples were then ultrasonically cleaned sequentially with acetone and deionized water for 5 min at room temperature. Prior to each experiment, all the samples were sterilized at 121 °C for 20 min.

### COM crystal preparation

Briefly, equal volumes of 10 mM calcium chloride (CaCl_2_) and 10 mM sodium oxalate (Na_2_C_2_O_4_) were mixed and equilibrated at 4 °C for 3 days and then harvested by centrifugation at 3000 rpm for 5 min. The supernatant was discarded, and the crystals were re-suspended in anhydrous methanol. After another centrifugation at 3000 rpm for 5 min, the methanol was discarded, and the crystals were dried at 37 °C overnight. The COM crystals were then decontaminated with UV light radiation for 60 min.

### Cell culture

Human ureteral epithelial cell lines (UECs), which were purchased from the Institute of Biochemistry and Cell Biology, Chinese Academy of Sciences, Shanghai, China, were cultured in DMEM medium (Hyclone, China), which was supplemented with 10% FBS (FBS, BI, USA) containing 100 U/ml penicillin and 100 µg/ml streptomycin (Logan, USA) in a water-saturated 5% CO_2_/95% air atmosphere at 37 °C. The cells were trypsinized and re-suspended in fresh media until 80% confluent. Before cells were treated with oxalate and COM, the culture medium was discarded and Ca^2+^-free DMEM, which also contained FBS, penicillin and streptomycin, was changed.

### Cell cytotoxicity assay

Cells were seeded in a 96-well plate at 3 × 10^3^cells/100 μl for each well and incubated for 8 h to allow attachment. For the treatment groups, the medium was replaced with 100 μl material extract as described previously^25^ and incubated for various time intervals (4, 12 and 24 h). The extract contained oxalate (0, 2, 5 or 10 mM) and COM crystals (200 μg/ml) to cause cell injury^41^. Cell viability was determined by MTS assay kit (Signalway Antibody, USA) according to the manufacture’s protocol. 10 μl MTS was added into the culture medium. After incubation for 4 h at 37 °C, the optical density of the medium was measured at 490 nm.

### Cell apoptosis

Cells were seeded on the samples and exposed to oxalate (2 mM) plus COM (200 μg/ml) crystals. After incubating for 24 h, cells were harvested, washed twice with ice-cold PBS, re-suspended in the dark with Annexin V-FITC and PI buffer (Sigma, USA), and finally analyzed on for apoptosis by flow cytometry. The cells were considered apoptotic when they were either Annexin V+/PI− (early apoptotic) or Annexin V+/PI+ (late apoptotic). All the flow cytometry data were analyzed by Mod Fit LT software.

### ELISA

The cells were seeded on the samples and exposed to DMEM with oxalate (2 mM) plus COM (200 μg/ml) for 24 h. The ratio of the surface area to culture medium volume was 3 cm^2^/ml. The supernatant was collected and used to measure the HA secretion into the medium. Meanwhile, proteins extracted from UECs were measured for the level of ROS. All the tests were conducted using an ELISA kit (Multi Science, China) strictly according to the manufacturer’s protocol.

### Western blot analysis

The cells were treated using the same ELISA method mentioned above and incubated for 24 h. The cells were then prepared for western blot analysis as described previously^42^. Immunoblot analyses were performed using CuZnSOD (1:1000, Santa), CD44 (1:800, Abcam), and GAPDH (1:2000, Abcam) antibodies. The optical density was quantified using ImageJ software.

### Real-time quantitative polymerase chain reaction (qPCR)

The cells were treated using the same protocol as that for ELISA described above. Total RNA was isolated using a TRIzol reagent (Invitrogen, USA) and was reversed and transcribed into complementary DNA (cDNA) using the PrimeScript 1st Strand cDNA Synthesis kit (TaKaRa, Japan) according to the manufacturer’s instructions. The gene expression was quantified using a real-time qPCR kit (SYBR Premix EX Taq, TaKaRa, Japan). The expression levels were normalized to the housekeeping gene GAPDH. The primers for the selected genes are shown in Table 2.

**Table 2:** Sequences of primer-pairs.

mRNA	Primer
F CuZnSOD	5′-CCTAGCGAGTTATGGCGACG-3′
R CuZnSOD	5′-CCACACCTTCACTGGTCCAT-3′
F CD44	5′-TGACAACGCAGCAGAGTAA-3′
R CD44	5′-CTTCGTGTGTGGGTAATGA-3′
F GAPDH	5′-AGAAGGCTGGGGCTCATTTG-3′
R GAPDH	5′-AGGGGCCATCCACAGTCTTC-3′

### Statistical analysis

Each sample was analyzed in triplicate, and the experiments were repeated three times. Data are expressed as the mean ± standard (SD). Student’s t-test and one-way analysis of variance (ANOVA) were used to compare the significant difference at *p* < 0.05.

## Electronic supplementary material

Supplementary files
