# Supplementary material for: Cu-bearing stainless steel reduces cytotoxicity and crystals adhesion after ureteral epithelial cells exposing to calcium oxalate monohydrate
Source: Sci Rep. 2018 Sep 20;8:14094. doi: 10.1038/s41598-018-32388-0 (PMC6148291; doi:10.1038/s41598-018-32388-0)
Supplement: Supplementary file 1 — Supplementary files [file 41598_2018_32388_MOESM1_ESM.doc]

**Cu-bearing stainless steel reduces cytotoxicity and crystals adhesion after ureteral epithelial cells exposing to calcium oxalate monohydrate**

Zhiqiang Cao, Jing Zhao, KeYang

**Supplementary Figures**


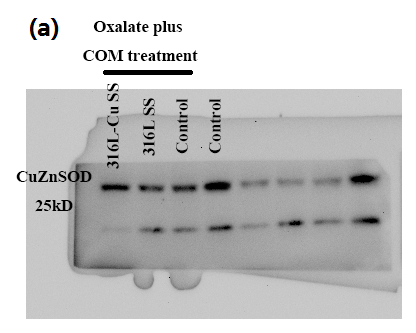

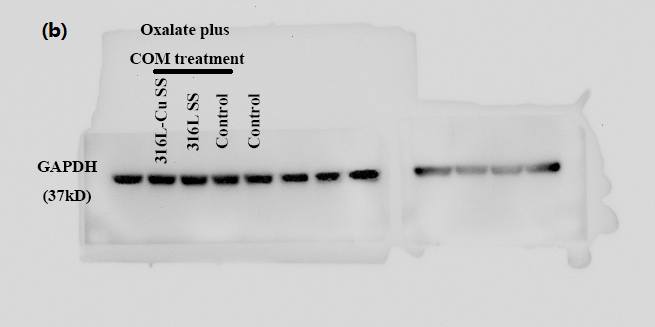


**Figure S1.** Raw data of the Western blotting experiments presented in Figures 3. The bands on the blotting membranes were visualized by chemiluminescence (see Methods section for further details). In all cases the lanes are displayed over the entire length of the blotting membrane. The blotting membrane did not always cover the whole gel in order to save material. Loading with sequential antibodies is indicated in the figure by panel arrangement from left to right. The figure not all lanes are shown because for the samples of interest only part of the gel was used. The remaining slots were filled with pilot samples unrelated to this work. After stripping, the blotting membrane was incubated with **(a)** anti-CuZnSOD and with **(b)** anti-GAPDH, respectively. The dilution rate of CuZnSOD with PBST was 1:1000, and that of GAPDH was 1:2000. The CuZnSOD antibody was from Santa, and GAPDH antibody was from Abcam.


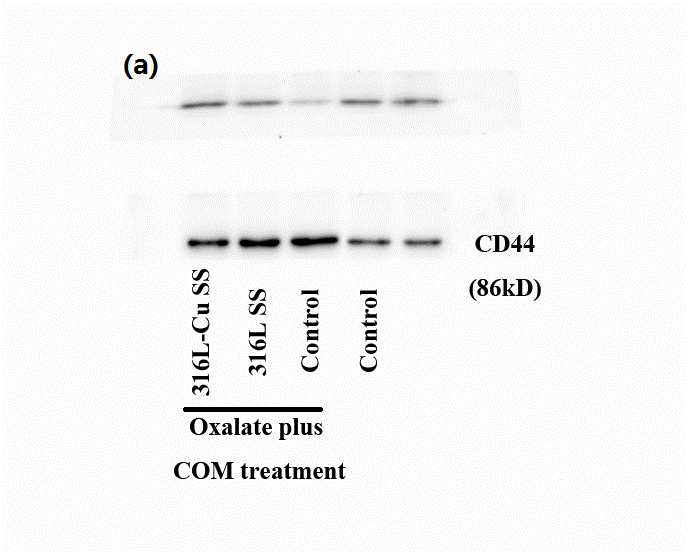

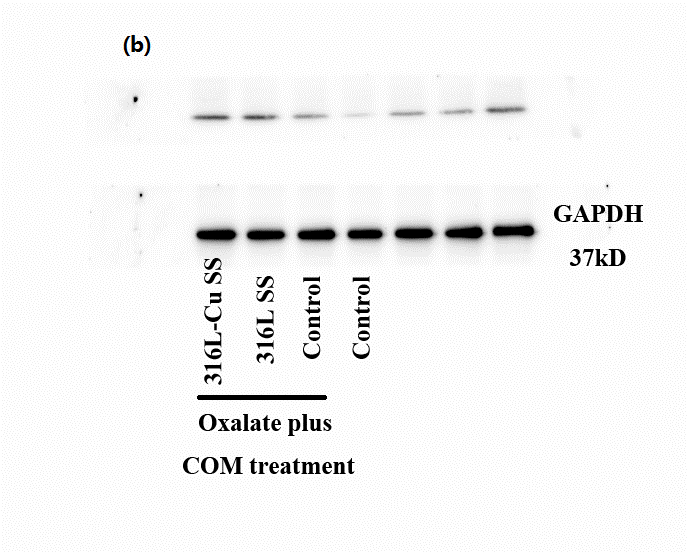


**Figure S2.** Raw data of the Western blotting experiments presented in Figures 5. The bands on the blotting membranes were visualized by chemiluminescence (see Methods section for further details). In all cases the lanes are displayed over the entire length of the blotting membrane. The blotting membrane did not always cover the whole gel in order to save material. Test and control antibodies were always incubated with the same blotting membrane; the membrane was stripped before incubation with the next antibody. Loading with sequential antibodies is indicated in the figure by panel arrangement from left to right. The figure not all lanes are shown because for the samples of interest only part of the gel was used. The remaining slots were filled with pilot samples unrelated to this work. The blotting membrane was first incubated with anti-CD44 and, after stripping, with anti-GAPDH as loading control. The dilution rate of CD44 with PBST was 1:800, and that of GAPDH was 1:2000. The antibodies were all from Abcam.
